# Validating a Web-based Diabetes Education Program in continuing nursing education: knowledge and competency change and user perceptions on usability and quality

**DOI:** 10.1186/2251-6581-13-70

**Published:** 2014-06-24

**Authors:** Marzieh Moattari, Elham Moosavinasab, Mohammad Hossein Dabbaghmanesh, Nahid ZarifSanaiey

**Affiliations:** 1 Faculty of Nursing and Midwifery, Shiraz University of Medical Sciences, Zand Street, Shiraz, Iran; 2 Endocrinology and Metabolism Research Center, Shiraz University of Medical Sciences, Shiraz, Iran; 3 Center of Excellence for Electronic Learning in Medical Sciences, Shiraz University of Medical Sciences, Zand Street, Shiraz, Iran

**Keywords:** Diabetes, Web-based Education, Evaluation, Nurses, Continuing Nursing Education

## Abstract

**Background:**

Nurses as the members of health care professionals need to improve their knowledge and competencies particularly in diabetes mellitus through continuing nursing education programs. E-learning is an indirect method of training that can meet nurses’ educational needs. This study is aimed at validating a web-based diabetes education program through measurement of nurses’ knowledge and clinical competency in diabetes and nurses’ perception about its usability and quality.

**Methods:**

This Quasi-experimental research was conducted on a single group of 31 nurses employed in hospitals affiliated with Shiraz University of Medical Sciences. We used a 125 MCQ knowledge test and Objective Structured Clinical Exam (OSCE) to measure knowledge and clinical competency of nurses in diabetes before and after intervention. A Learning Management System (LMS) was designed to provide educational content in the form of 12 multimedia electronic modules, interactive tests; a forum and learning activities. Nurses were trained for two months in this system after which the post-test was administered. Each nurse completed two questionnaires for measurement of their perceptions on usability and quality. We used descriptive statistics for demographic and descriptive data analysis. Paired t-test was used to compare pre- and post-data using SPSS.

**Results:**

The findings showed significant differences in knowledge scores (p < 0.001), total score of clinical competencies (p < 0.001), and all ten assessed clinical competencies. The range of ratings given by participants varied on the six usability variables of Web-based training (2.96-4.23 from 5) and eight quality variables of Web-based training (3.58-4.37 from 5).

**Conclusion:**

Web-based education increased nurses’ knowledge and competencies in diabetes. They positively evaluated Web-based learning usability and quality. It is hoped that this course will have a positive clinical outcomes.

## Introduction

Continuing Nursing Education (CNE) is a felt need for nurses in many areas such as diabetes mellitus. Diabetes mellitus as a costly, chronic disease, [1] affects increasing numbers of people worldwide. Its prevalence among adults (20–79 years) will increase to 7.7%, affecting 430 million adults by 2030 [2]. The prevalence of type 2 diabetes mellitus (T2DM) in Iran by 2025 is estimated at 6.8% [3].

Patient with diabetics are hospitalized more than the general population. While a holistic approach to care (by experts) is needed to diminish the effects of this complex health problem [4], there are some studies indicating a lack of diabetes knowledge among nurses. For example Uding et al. observed that over half of the participant nurses lacked up-to-date knowledge about diabetes and a percentage had never participated in continuing diabetes education programs [5]. In another study, participants scored a mean score of 64% on a basic knowledge test which indicated a lack of knowledge about diabetes [6]. Therefore a nursing continuing education program is essential that emphasizes diabetes and health promotion [4].

Continuing education programs include various venues such as seminars, conferences, workshops, short courses, and professional medical programs. Most require participants to be present [7]. Nurses however may not be able to participate in such kind of programs because of their shifts, and work pressures [8].

Virtual and e-learning methods can provide new opportunities for nurses to meet their educational needs [9]. E-learning is a teaching strategy characterized by mutual interaction and active participation in the learning process [10]. Those who participate in e-learning have access to 24-hour training, can study on their own pace and do not need to travel for attendance in in-person classes. E-learning does not interfere with work schedules and the time required to learn is reduced by 25%-30%. E-learning saves time and cost for training teachers and educational activities. Training materials are developed only once, yet used numerous times in different locations [11]. There are different ways to provide E-Learning but those which provide interaction among the learners as well as learners with teachers are more appropriate in producing learning. Learning management system (LMS) provides educational content and allows for interaction and collaboration through numerous discussions, news, forums, blogs and tests. LMS leads to collaborative learning. Teachers can electronically assess, rank and record students’ progress according to their basic information [12].

Studies have assessed the achievement of learning goals by researching e-learning and its impact in different ways. For example in one study nurses’ attitudes toward Web-based learning modules and their prior learning experiences was evaluated. Participants positively evaluated all relevant factors such as general assessment of educational outcomes and achieving learning objectives, level of support and learning materials [13]. This study did not research the effect of education on knowledge and capabilities of individuals. Another study analyzed Web-based learning experiences of nursing managers’ ideas about the usefulness of information technology in improving professional health care through interviews. Participants stated that Web-based training was a modern learning method [14]. Quality control of such modern learning particularly meeting the learning objectives, formative and summative evaluation has been highlighted in another study [15]. Eaton used PowerPoint files as modules to evaluate nurses’ knowledge and skills. The Diabetes Self Report Tool (DSRT), a self-report test that measures diabetes knowledge and nursing skill was used. Education positively impacted knowledge and nursing skills despite one-way interaction and feedback [16]. In another study the effectiveness of a Web-based computer training modules was approved based on measurement of nurses’ knowledge, attitude and skills. However nurses skills was measured by questionnaires which reflects nurses’ beliefs about their skills [17]. Another study compared the effects of lectures and computer education on nurses’ knowledge. Regardless of the training type, knowledge scores increased following training according to the Diabetes Basic Knowledge Test (DBKT) [1].

Miller provided a framework for assessing levels of clinical competence [18]. However none of these studies used an objective method to examine nurses’ capabilities. Based on Miller’s framework (pyramid) there are four levels of competency: knowing, knowing how, showing and doing. The lower two levels measure cognition and the upper two measure behavior. Miller’s pyramid has been used for over twenty years as a framework to evaluate clinical skills at different levels [19]. Objective Structured Clinical Examination (OSCE) is an objective, reliable test that measures ability by showing the status of the learners in show how level of the Miller pyramid. The feasibility of OSCE to assess nursing students’ skills was approved [20].

While patients with diabetes were targets for Web-based training in Iran [21] nurses did not benefit from these programs. A search with the key words “Learning - Nurse and Diabetes”, in IranMedex and Magiran provided no evidence of Web-based CNE program in Iran. Considering the limitations and challenges associated with current traditional educational methods applied in our Iranian CNE programs and the shortcomings of the above mentioned literature, we decided to design a Web-based diabetes CNE program and evaluate its effectiveness by measuring the possible changes in nurses’ knowledge and competencies. Also nurses’ perceptions about usability and quality of the program are assessed.

## Materials and methods

This single group quasi-experimental study evaluated the impact of Web-based education on nurses’ knowledge and competency of types 1 and 2 diabetes. Pre- and post-tests, and nurses’ perceptions about its usability and quality were studied. Considering a small effect size (delta 0.3) and 95% confidence level the sample size was determined to be 58. We chose five teaching hospitals affiliated with Shiraz University of Medical Sciences with Internal, Surgical, Cardiovascular Surgery and Endocrine wards.

From 100 nurses eligible to attend, we invited 60 to participate according to stratified random sampling. Of these, 45 agreed to participate and 31 completed the study. Inclusion criteria included: bachelor’s degree or higher; employment as a nurse in the mentioned hospital wards; 8–15 years work experience; no concurrent participation in a diabetes education program; no participation in any diabetes CNE programs within the previous year; and willingness to participate. We sent pre-test announcements to offices of selected hospitals followed by invitation letters to all participants. Participants gave their informed consent, after which they completed a personal information form.

Data was collected using demographic data collection form, knowledge test, OSCE, and two questionnaires for measurement of nurses’ perceptions on usability and quality of Web-based education. We designed a knowledge test of 125 multiple choice questions according to objectives predetermined by a panel of experts. A five-member group of Medical-Surgical nursing professors in the Nursing faculty confirmed its validity. For reliability, a similar group of 20 nurses completed the knowledge test. Test reliability was confirmed by Kuder-Richardson 20 (kr = 0.81).

For competency assessment, we designed ten stations for the OSCE. Subjects for each station were selected according to the purposes of a T2DM education program by the International Diabetes Institute [22] and educational references [23-25]. We developed checklists to evaluate competencies in each of the ten stations. Participants were observed by experienced nurses familiar with principles of observation. All observers attended a briefing session regarding the related stations, checklists, and standard patients if applicable.

To maintain reliability, we established ten stations so that the length of the OSCE was 57 minutes. To measure inter-rater reliability, two raters observed nurses’ competencies in six of the ten stations that were selected randomly. The Pearson correlation between the scores of both raters was calculated. There was a bilaterally significant relationship and desirable reliability (0.01) in the six stations. Table 1 shows information regarding the duration per station, number of items observed (checklist) and inter rater reliability coefficient. OSCE was performed according to previous experience [20] and procedures for the pre- and post-tests were similar.

**Table 1:** OSCE stations profiles

**Station title**	**Item number**	**Duration**	**Reliability coefficient**
1. Foot examination	16	5 min	0.003
2. Face to face education about foot care for prevention of diabetic Foot	11	5 min	0.003
3. Learning how to properly draw and mix insulin to diabetic Patient	15	7 min	
4. Learning how to properly self inject insulin to diabetic patient	13	5mim	
5. Learning sick days rules for diabetic patient	11	7 min	<0.001
6. Training important points of exercise to diabetic patient	14	7 min	<0.001
7. Measure of blood glucose by glucometer	15	4 min	
8. Teaching of important nutritional points to diabetic patient	9	7 min	<0.001
9. Diabetic patient education based on health belief model	5	8 min	<0.001
10. Response to diabetic patients FAQ	Open end question	2 min	

We used a 35-item questionnaire to assess participants’ attitudes toward usability of Web-based education by evaluating computer use, computer learning, distance learning, course support, learning outcome, utility of the material, and overall course evaluation. The questionnaire initially developed by Wilkinson et al. [13], had a Cronbach’s alpha coefficient of 0.84.

The 49-item questionnaire that evaluated the quality of the Web-based educational program included content, learning and support, visual design, navigation, accessibility and intra-activity, self-assessment, ability and motivation to learn. Zaharias designed this questionnaire. Its validity and reliability was confirmed by numerous researches [26-28].

Both study questionnaires were translated into Persian by two separate experts and back-translated into English. Finally two English versions were compared by qualified bilingual experts to ensure validity. Experts in training and computer-based programs verified the appropriateness of the questionnaires’ contents. The reliability of the questionnaires was assessed by participants in another e-learning program. Test-retest correlation coefficients for the attitude toward usability of Web-based training questionnaire and the quality of Web-based training questionnaire scores in a two-week interval were 0.81 and 0.84, respectively.

### Research intervention

A LMS was chosen for this research by the Center for Continuing Education and Excellence in Education at Shiraz University of Medical Sciences. Using their expertise “Docebo” learning management system designed to address *e-cne.sums.ac.ir*.

### Design and implementation of the LMS

The training course for diabetes was identified. Educational goals and objectives were set. Educational outlines were established based on those goals and objectives. Latest available sources and educational programs on diabetes topics presented in 2010 by the International Diabetes Federation (IDF) [22] and expert opinions (endocrinology and metabolism specialists and experienced nurses) about appropriateness of educational outlines were considered in this stage. The contents were chunked into multiple shareable content objects (SCOs).We defined instructional strategies for each SCO.

The course consisted of 12 electronic modules designed according to these instructional strategies. Each module was delivered using animated slides, images, video sequences, forms, tables and charts with synchronized narrations. Each module included a self-assessment pre-test, general purpose, multimedia educational content, assignments, self-evaluation post-test, glossary and summary. A list of sources used in the educational content was provided.

### Features and capabilities of the Learning Management System (LMS)

The LMS included the forum, ability to send and receive both private and public messages, self-assessment tests, scores and recorded test results, allocation of the test scores, ability to view the contents in the desired frequency, ability to view the number of people online and the total hours worked in the system at any time participants were online. To increase motivation, topics such as public comments and learning activities were discussed in the forum with the intent to share users’ ideas and opinions. During the program, forum participants raised various questions such as how to inject insulin, dosage adjustments of insulin during fasting and following surgery. Participants received appropriate responses.

The LMS allowed researchers to monitor users’ performance capabilities by conducting a review of the modules, date and time of participants’ entry and exit from the system, type of activities each time participants logged on and their ranking.

### Implementation of the educational program

Each participant received a username and password sent via cell phone message. The LMS address and electronic systems guidelines that included complete details were sent to participants’ e-mails. Participants received telephone support. The study time allocated to each module averaged 5 days (total:60 days)for the twelve modules.

Subjects initially answered pretest self-assessment questions. By providing feedback (grades), they were aware of gaps in their knowledge for each module (self-evaluation). Subsequently participants completed performed learning activities associated with that module. The learning activities included essay questions, scenarios with multiple choice questions, referral to other diabetes educational sites and discussion about questions raised in the forum. For example, for the practice of risk factors related to diabetes, the address of a reputable site (http://www.worlddiabetesday.org/bluecircletest) was given to participants as a link where they could enter personal information and receive their diabetes risk factors. Results were discussed in the forum.

Participants answered the modules’ post-test questions. In addition to receiving the final score they were given the correct answers, as feedback. Each step was a prerequisite for the next step; participants were unable to continue without completing the previous step. In order to achieve the goals in the psychomotor domains, we used online training videos such as “How to check blood sugar with a glucometer” and “How to use insulin pens”. Addresses of other diabetes educational sources were links on the website.

### Scoring and data analysis

Descriptive statistics analyzed demographic data, Pearson correlation test was used to determine the inter-rater correlation and the paired t-test compared data before and after intervention by SPSS (version 17.0, Chicago, IL, USA) Separate scores in the knowledge test for each of the 12 training modules were obtained according to a grading scale of 2 with the intent to view the scores from each module in a unified scale. According to the number of training modules each assigned a value of 2, we calculated the mean knowledge score on a scale of 24. Higher scores indicated higher levels of diabetes knowledge. Competency scores for each OSCE station were calculated based on a scale of 2; the total score of the OSCE was calculated based on 20. Scores of the Web-based education usability perception questionnaire, in each of the sections were calculated according to a scale of 1 (never) to 5 (always) and 1 (strongly disagree) to 5 (strongly agree). Eight options of this questionnaire were negatively-keyed options phrased such that agreement with them represented a relatively low level of understanding of Web-based instruction. We used reverse scoring for these options.

The Web-based quality questionnaire contained 49 questions for measurement of eight variables, each assigned a point value of 1 (strongly disagree) to 5 (strongly agree). Questions that contained the option “not applicable”, had no assigned score.

In two recent questionnaires, the total score obtained in each section was divided by the number of questions for that score (mean rating). Thus, each individual score in each section ranged from 1 to 5. In each section, higher scores indicated greater perception of usability and quality of Web-based education.

### Ethical considerations

Institutional Review Board (IRB) approval for the study was obtained from the Ethics Committee of University of Medical Sciences (ECSUMS). Written consent was obtained from each nurse. The purpose of study, voluntary participation, and freedom to discontinue at any time was reviewed with participants prior to their participation. Participants were assigned study code numbers to ensure their anonymity. All participants received a CNE certificate for their attendance in the program. They also were assured that their scores on both knowledge and competency exams are used just for statistical analysis. Name of the institution where the work was performed: Shiraz University of Medical Sciences (SUMS), Shiraz, Iran.

## Results

Participants in the Web-based diabetes education program were all female nurses, 25–50 years old (mean: 36.29 ± 6.19). Of participants, 71% were married. Experience ranged from 8–15 years (average: 12.44 ± 5.02).

The mean baseline diabetes knowledge score was 11.26 ± 0.834 from 24. This increased to 17.47 ± 0.607 after Web-based education (p < 0.001). Table 2 compares the mean total scores for each separate training module before and after Web-based education. Results of paired t-tests in all modules, except the foot ulcers module, showed significant changes in knowledge scores.

**Table 2:** Comparison of nurses’ diabetes knowledge mean score as a total and in each module separately, before and after the web based education

**Educational modules**	**Prior education mean ± SD**	**Post education mean ± SD**	**p-value**
Diabetic neuropathy	1.30 ± 0.372	1.85 ± 0.200	<0.001
Diabetic foot	1.24 ± 0.313	1.46 ± 0.338	0.062
DKA&HHS	1.15 ± 0.330	1.43 ± 0.300	0.004
Hypoglycemia	1.20 ± 0.314	1.62 ± 0.264	<0.001
Macrovascular diseases	1.13 ± 0.363	1.53 ± 0.263	<0.001
Principals of patient education	0.987 ± 0.434	1.47 ± 0.281	<0.001
Diagnosis, classification and prevention in diabetes	0.879 ± 0.304	1.31 ± 0.361	0.002
Insulin	0.968 ± 0.281	1.50 ± 0.259	<0.001
Nutrition and exercise in diabetes	0.889 ± 0.487	1.49 ± 0.245	<0.001
Diabetic retinopathy	0.725 ± 0.240	1.27 ± 0.177	<0.001
Diabetic nephropathy	0.658 ± 0.294	1.24 ± 0.360	<0.001
Oral anti-diabetes medication	0.675 ± 0.317	1.38 ± 0.296	<0.001
Total score (from 24)	11.26 ± 0.834	17.47 ± 0.607	<0.001

Nurses’ competency score prior to Web-based education was 9.13 ± 1.60 from 20 which increased to 15.27 ± 1.50 after intervention (p < 0.001). The total mean score of competency in diabetes and competency scores for each station are shown in Table 3.

**Table 3:** Comparison of nurses’ diabetes competency mean score as a total and in each station separately, before and after the web based education

**Station title**	**Before education mean ± SD**	**After education mean ± SD**	**p-value**
1. Foot examination	0.87 ± 0.297	1.45 ± 0.289	0.001
2. Face to face education about foot care for prevention of diabetic foot	0.59 ± 0.369	1.22 ± 0.340	<0.001
3. Learning how to properly draw and mix insulin to diabetic patient	1.25 ± 0.343	1.66 ± 0.312	0.001
4. Learning how to properly self inject insulin to diabetic patient	1.23 ± 0.384	1.58 ± 0.223	0.003
5. Learning sick days rules diabetic patient	0.40 ± 0.219	0.78 ± 0.403	0.011
6. Training important points of exercise to diabetic patient	0.60 ± 0.143	1.33 ± 0.359	<0.001
7. Measure of blood glucose by glucometer	1.20 ± 0.508	1.91 ± 0.886	<0.001
8. Education of Important nutritional points to diabetic patient	0.85 ± 0.285	1.88 ± 0.187	<0.001
9. Diabetic patient education based on health belief model	0.68 ± 0.279	1.58 ± 0.196	<0.001
10. Response to diabetic patients FAQ	1.42 ± 0.759	1.89 ± 0.416	0.023
Total score (from 20)	9.13 ± 1.60	15.27 ± 1.50	<0.001

Table 4 shows the mean ratings given to the usability and quality of Web-based education. Overall assessment of course variable had the highest mean rating and the computer use variable was the lowest rated among usability variables. For quality variables, the highest mean rating was self-assessment and the ability to learn and the accessibility variables were the lowest.

**Table 4:** Mean ratings given to usability and quality of Web-based program

**Usability variables**	**Mean ± SD**
1. Computer use	2.96 ± 0.90
2. Computer learning	3.54 ± 0.724
3. Distance learning	3.42 ± 0.480
4. Overall course evaluation	4.23 ± 0.588
5. Course support	3.50 ± 0.954
6. Utility of material	4.13 ± 0.496
**Quality Variables**	
1. Content	4.37 ± 0.427
2. Learn & support	4.01 ± 0.663
3. Visual design	4.29 ± 0.679
4. Navigation	4.13 ± 0.706
5. Accessibility	3.58 ± 1.26
6. Interactivity	4.05 ± 0.735
7. Self assessment & learnability	4.37 ± 0.623
8. Motivation to learn	4.28 ± 0.615

## Discussion

This study evaluated the impact of Web-based diabetes education on knowledge and competency. We also assessed nurses’ perception about usability of Web-based education and its quality. There was a significant increase in total mean scores of nurses’ diabetes knowledge and competency after Web-based education. Participants positively evaluated Web-based education.

In a similar study, the mean score for DSRT in nurses’ knowledge and skills increased following training [16]. The increased score in the present study (6.21/24) was higher compared to Eaton’s study, which showed an increase of 1.35/88 (knowledge). This discrepancy could be due to differences in participants’ characteristics, nurses’ knowledge base and training between the studies. The electronic module in Eaton’s study contained a PowerPoint file uploaded in an educational website with intranet connections and no possibility of interaction or feedback as this was a one-way style of learning.

Wright [1] researched the effect of conventional and computer education on 175 nurses and their diabetes knowledge as evaluated by DBKT. Knowledge scores in both groups increased, which supported the current study results. Wright’s study used an online virtual learning environment (Web-CT) with educational content in a PowerPoint file [1]. Other studies confirmed the effectiveness of electronic education in physicians and dentistry students [29-33].

The current study results showed that Web-based learning significantly increased competencies in all areas of diabetes. Other studies did not evaluate nurses’ competencies in different areas of caring for diabetics.

The impact of Web-based learning to enhance nurses’ skills in some areas as evaluated within non-objective methods was supported. A single-group study placed their instructional content about evidence-based learning in three computer training modules on a Web site. At two weeks following completion of the training modules, evidence-based nursing skills were assessed with a written test. The results supported improvement in the level of nursing skills [17].

The positive impact of electronic and Web-based learning on enhancing nurses’ competencies in the current study can be attributed to the learning path provided. For example to reach the goals of the psychomotor domains, we used online videos such as “How to use a glucometer” and “How to use an insulin pen”.

Nurses’ perception about usability of Web-based education had a mean rating for all variables of > 2.5. Participants positively evaluated overall course evaluation, course support and utility of the materials. The lowest mean rating was the computer use variable. Computer knowledge and access to hardware and software facilities effectively increase usability capacity of educational courses [34]. One study showed a significant relationship between computer use and participants’ attitudes to internet-based continuing education [35]. A direct relationship exists between Internet use and positive attitudes towards the subject [13]. Nurses’ ratings of computer learning and distance learning in this study were lower than Wilkinson’s study [13]. Participants in this study had little previous experience with computers. It can be concluded that to generate successful online continuing education courses for nurses, the use of computers and the Internet should be facilitated.

Users were satisfied with content quality of electronic modules in terms of composition and structure. The use of experts for distance education planning and development of electronic modules with properties listed in the method led to their positive views. The content, teaching and learning quality was an acceptable criterion for evaluating LMS [36]. User satisfaction is recognized as the main element of Web-based education quality [37].

The mean rating for Web-based learning and support was 4.01 from 5 which can be attributed to the use of e-mail, forum, and exchange of ideas and interactions between learners and the system administrator in the LMS. The use of scenarios and self-evaluation might explain this result. One study that evaluated a blended e-learning program for nurses found that only 28.2% of nursing students were fully satisfied with issues related to learner support. The reason for this dissatisfaction was the lack of opportunities for interaction between the learner, educator and other learners or lack of additional supports for electronic interaction. In that study both students and teachers expressed their satisfaction with the application of blended e-learning content and lesson plans [38].

Accessibility had the lowest mean rating. Nurses viewed accessibility to the diabetes LMS as difficult. The high volume of the video and audio-visual applications in electronic modules and use of low-speed Internet service limited quick access to the modules. The problems with accessing LMS were not unexpected because this type of education was not previously experienced by participants.

The mean rating for the interactivity item in the Web-based quality questionnaire was 4.05 from 5, which was considerable. The LMS used in this study was successful. The quizzes and learning activities associated with providing feedback, the placement of a number of learning activities in the forum, encouraging nurses to participate in discussions, and answering questions in interactive learning activities in the forum all created opportunities for improvement of knowledge and competencies. Private and group messaging, chat format and the presence of educational links were other strategies that increased interaction. Participants were aware of their needs and used the e learning environment as a tool for meaningful interaction. It has been emphasized that activities and training programs should be part of a meaningful, useful learning environment through providing opportunities for learners to apply their knowledge [15]. Experience shows that interactive computer modules are a useful supplement to assist learners with understanding difficult concepts in clinical medical education [39].

Self-assessment and the ability to learn had the highest mean rating (4.37) among items of this questionnaire. The diabetes Web-based program provided adequate opportunities for participants’ self-assessment by using pre-and post-tests for self-evaluation in each module in addition to program learning activities.

The mean ratings of the Web-based quality questionnaire were >80% of the possible score, except for one item. We concluded that the quality of the created LMS in this study was satisfactory. Another study determined that user satisfaction was the most important contributor to the evaluation of a program’s success [36].

Despite the effectiveness of the web based learning in the current study and providing CNE credit to the participants, nearly half of the volunteers chose not to participate. The program’s extensiveness, implementation of the knowledge test and OSCE for pre- and post-tests, as well as barriers to the use of computers and internet might be causes for lack of participation. Therefore, the need to adopt effective policies and mechanisms to encourage participation in these programs should be considered.

In conclusion this study showed that Implementation of Web-based education is feasible and leads to increased knowledge and competency of nurses. Nurses attending the program showed their satisfaction with the usability and quality of the program.

## Abbreviations

OSCE: Objective Structured Clinical Exam; LMS: Learning Management System; MCQ: Multiple Choice Question; CNE: Continuing Nursing Education; DSRT: Diabetes Self Report Tool; T2DM: Type2 diabetes mellitus; SUMS: Shiraz University of Medical Sciences, Shiraz, Iran.

## Competing interests

The authors declare that they have no competing interests.

## Authors’ contributions

MM devised the concept for the study, developed the study design, supervised data collection and analysis, drafted the manuscript, and was involved in study coordination and manuscript revision. EM collected data, ran the study intervention, was involved in the conception of the study, performed the analyses and drafted the manuscript. MHD contributed to the study design and intervention. NZS contributed to the design of LMS. All authors read and approved the final manuscript.
